# Association Between Area Deprivation Index and Melanoma Stage at Presentation

**DOI:** 10.3390/cancers17172772

**Published:** 2025-08-26

**Authors:** Rachael Cowan, Elizabeth Baker, Mohammad Saleem, Victoria Jiminez, Gabriela Oates, Lucia Juarez, Ariann Nassel, De’Travean Williams, Nabiha Yusuf

**Affiliations:** 1 UAB Heersink School of Medicine, University of Alabama at Birmingham, Birmingham, AL 35233, USA; 2 Department of Anesthesiology and Perioperative Medicine, University of Alabama at Birmingham, Birmingham, AL 35249, USA; 3 Department of Dermatology, University of Alabama at Birmingham, Birmingham, AL 35233, USA; 4 Department of Medicine, UAB Heersink School of Medicine, University of Alabama at Birmingham, Birmingham, AL 35294, USAlucia@uab.edu (L.J.); 5 Lister Hill Center for Health Policy, School of Public Health, University of Alabama at Birmingham, Birmingham, AL 35233, USA; 6 Department of Natural Sciences, Stillman College, Tuscaloosa, AL 35401, USA

**Keywords:** Area Deprivation Index, neighborhood disadvantage, social determinants of health, health disparities, melanoma, skin cancer, stage at diagnosis, insurance status

## Abstract

**Background/Objectives**: Later-stage melanoma at diagnosis is associated with increased mortality. Health care access, socioeconomic status, and neighborhood-level factors likely influence stage at presentation. This study aimed to examine whether neighborhood disadvantage, as measured by the Area Deprivation Index (ADI), is associated with later-stage melanoma diagnosis. **Methods**: We conducted a cross-sectional analysis of a retrospective cohort of 941 patients diagnosed with melanoma at a large academic medical center between 2010 and 2019. Residential addresses were geocoded and linked to ADI and rurality data. Covariates included race, ethnicity, age, gender, and insurance status. Multivariable logistic regression models with robust standard errors clustered at the census tract level were used to assess associations with melanoma stage at diagnosis. **Results**: Of 941 patients (63% male, 92.8% non-Hispanic White, mean age 64 years), 432 (46%) were diagnosed with late-stage melanoma. Mean ADI was higher among late-stage cases (5.4) compared to early-stage cases (3.3) (*p* < 0.001), even after adjustment for covariates. Non-Hispanic White race, private insurance, older age, and urban residences were associated with earlier stage at diagnosis. Racial disparities were attenuated after adjusting for ADI, with no significant interaction between race and ADI. **Conclusions**: Neighborhood disadvantage is significantly associated with later-stage melanoma diagnosis and contributes to observed racial and socioeconomic disparities. These findings highlight the need for targeted educational interventions and health policy initiatives to reduce late-stage melanoma diagnoses in vulnerable populations.

## Simple Summary

Melanoma is a common and potentially life-threatening skin cancer and prognosis is closely tied to the stage at which it is diagnosed. Individuals residing in socioeconomically disadvantaged neighborhoods may face structural barriers to timely diagnosis, contributing to disparities in cancer outcomes. In this study, we analyzed melanoma cases from a large academic medical center to investigate whether neighborhood-level disadvantage, measured by the Area Deprivation Index (ADI), was associated with later-stage diagnosis. We also explored whether ADI and insurance status mediated observed racial disparities. Our findings demonstrate that a higher neighborhood disadvantage is significantly associated with advanced-stage melanoma at presentation, independent of individual demographic and socioeconomic factors. These results underscore the importance of addressing social determinants of health in cancer prevention efforts. Interventions targeting high-risk communities may improve early detection and reduce disparities in melanoma outcomes.

## 1. Introduction

Melanoma is a severe form of skin cancer that predominantly affects non-Hispanic White patients, yet minority populations suffer disproportionately from late-stage diagnoses and poorer prognoses [1,2,3]. Survival rates for melanoma are closely tied to the stage at diagnosis, with delayed detection significantly reducing the likelihood of successful treatment [4,5,6,7]. Social determinants of health (SDOH), including socioeconomic status (SES), educational attainment, access to healthcare, and rural residence, negatively affect melanoma prognosis, and factors associated with access and socioeconomic status likely contribute to racial disparities [3,8,9,10,11].

Despite melanoma’s higher overall incidence among individuals with higher SES, poorer prognosis and late-stage diagnoses are correlated with lower SES [4,6,8,9,11,12,13]. This paradox underscores the complexity of SDOH and their multifaceted impact on health outcomes. Furthermore, this paradox may be directly related to the lower likelihood for racial minorities to be diagnosed, the relationship between SES and race in the United States, and the worse prognosis among those, once diagnosed. Recent SDOH research has incorporated measures like the Area Deprivation Index (ADI), which aggregates variables related to income, education, employment, and housing quality at the Census Block Group level and are standardized to either the state or national level [14,15,16]. State ADI scores, ranging from 0 to 10, provide a quantifiable metric of deprivation, with higher scores indicating greater levels of deprivation. The ADI evaluates neighborhood-level socioeconomic disadvantage, offering valuable insights into the social context that profoundly affects individuals’ access to and benefits from health resources [14,15,16].

A few studies have explored the association between area-based measures and melanoma outcomes [9,10,17,18,19], though these studies used various methods to calculate neighborhood disadvantage, which can limit generalizability. Also, these studies were conducted in Florida, Texas, California; Alabama is unique from these other states given that a much higher percentage of the population lives in rural areas [20]. In the 2010 Census, 41% of Alabama’s population resided in rural areas compared to 5% for California, 9% for Florida, and 15% for Texas [20]. A few recent studies have also explored this relationship. Moncayo et al. explored the association between ADI and melanoma tumor thickness in the Veterans Health Administration setting, while Goyal et al. used the Social Vulnerability Index as a measure of neighborhood disadvantage [21,22]. Unlike these studies, our work examines the stage of diagnosis in a general academic medical center population and uses the Area Deprivation Index as the measure of neighborhood disadvantage.

This study aims to bridge the gap in understanding the relationship between social vulnerability and melanoma outcomes by utilizing ADI. By geocoding patient residential addresses and linking them to ADI and healthcare access data, including rurality and insurance, we aim to identify whether more deprived neighborhoods translate into higher risk for delayed melanoma diagnosis within the UAB health system cohort from 2010 to 2019. Special attention is given to racial disparities, examining whether neighborhood SES and health insurance account for inequities in melanoma diagnosis stages and whether the association of ADI varies by race/ethnicity.

Understanding these dynamics is crucial for developing targeted education programs and policy recommendations to prevent late-stage melanoma diagnoses, particularly in vulnerable communities [23]. By addressing the socioeconomic inequities in melanoma outcomes, this research aims to contribute to more equitable healthcare and improved survival rates for all melanoma patients.

## 2. Materials and Methods

Data on patients with melanoma of the skin diagnosed between 2010 and 2019 was obtained from electronic medical records at UAB. Patient residential addresses including street address, city, state, and zip code were collected at the time they were diagnosed with melanoma and were geocoded using Esri ArcGIS Pro version 3.4. Geocoded addresses were then linked to relevant indices by performing a spatial join in ArcGIS Pro version 3.4 and using the 2010 Census block group or Census tract.

**Figure 1:**
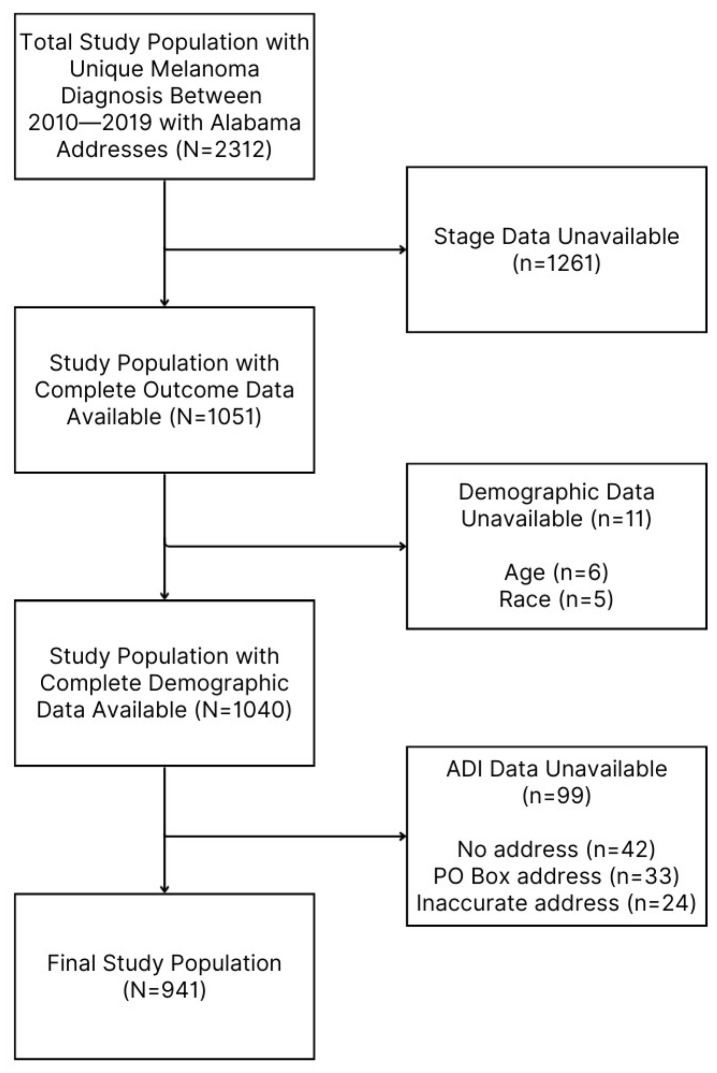
Study population selection flow chart. ADI, Area Deprivation Index; PO, post office.

Only patients with addresses in Alabama were included. Addresses that could not be accurately geocoded (e.g., missing information, PO Box, or incorrect address) were excluded. After removing cases with missing stage data, duplicate entries, and incomplete covariate data, the final data set included 941 patients (Figure 1).

### 2.1. Outcome

The primary outcome variable was stage of cancer at diagnosis, measured as stages 1, 2, 3, and 4. For this analysis, melanoma staging was dichotomized into early-stage (stages 1–2) compared to late-stage (stages 3–4) categories. This classification reflects clinically significant differences in survival rates between these groups and facilitates the interpretation of findings [5]. Although dichotomization may result in the loss of some information, it aligns with the study’s aim to evaluate broad disparities in outcomes across clinically meaningful categories.

### 2.2. Predictor

ADI is an existing composite measure of socioeconomic disadvantage based on 17 variables from the domains of income, education, employment, housing, and family structure collected by the American Community Survey (ACS) and aggregated into Census block groups [14]. The ADI is a reliable metric for neighborhood socioeconomic conditions, previously used with several pediatric cohorts [14,24,25,26,27,28]. State-specific ADI scores (1–10 scale, with higher scores indicating a higher disadvantage) for geocoded patient addresses were obtained from the Neighborhood Atlas [15,16]. This study uses the 2015 ADI model, which aligns with the midpoint of our study period, to provide a representative measure of neighborhood conditions.

Race/ethnicity was measured as non-Hispanic White, non-Hispanic Black, and a residual other race category.

### 2.3. Covariates

Race/ethnicity, urbanicity, age, gender, marital status, and health insurance were included as covariates. Urbanicity is measured using Rural-Urban Commuting Area (RUCA) and is based on Census tracts. An area is defined as urban if RUCA equals 1 and non-urban otherwise. Age was measured by birthdate and admission date. Health insurance status was measured as private insurance compared to those with public insurance or self-pay.

### 2.4. Statistical Analysis

Descriptive statistics, including means, standard deviations, percentages, and 95% confidence intervals, were calculated for all study variables and separately by stage at diagnosis. Multivariate analysis was conducted using logistic regression with robust standard errors clustered at the census tract. Model 1 included race/ethnicity and established race/ethnicity differences in stage at diagnosis. Model 2 included ADI and assessed the extent that ADI accounts for race/ethnic differences documented in Model 1. Model 3 included the covariates age, gender, marital status, urban, and health insurance and assessed the extent that ADI was an independent predictor of cancer stage. Lastly, Model 4 examined whether the association between ADI and stage varied by race/ethnicity by including an interaction between the two variables. Results are presented both as odds ratios and predicted probabilities to capture both the significance and the magnitude of later-stage at diagnosis by race/ethnicity [29].

## 3. Results

### 3.1. Patient Demographic Characteristics

Table 1 displays the means and percentages of the study variables by stage at diagnosis. A total of 941 patients were found to be diagnosed with a melanoma at the UAB health system between 2010 and 2019 with valid residential addresses to be included in the study. Of these, 509 patients were diagnosed at early-stage and 432 were diagnosed at late-stage. Of the full sample, 92.8% are non-Hispanic White, 63% are male, 42.3% have private insurance, and 45.9% of participants were diagnosed with melanoma at a later-stage. Of the 432 participants who were diagnosed at a later-stage, 89.4% are non-Hispanic White, 66.2% are male, and 35.6% have private insurance.

### 3.2. Differences in Stage at Diagnosis by ADI, Race/Ethnicity, Insurance, Rurality, and Age

Significant differences in stage at diagnosis were observed across multiple sociodemographic factors. The mean ADI for individuals diagnosed at a later-stage was 5.4, compared to 3.3 for those diagnosed at an earlier stage (*p* < 0.001). This was also demonstrated when examining the ADI quartiles. Those in the lowest ADI quartile (least disadvantaged) were more likely to have an earlier-stage diagnosis compared to those diagnosed at a later-stage (36% compared to 11.8%; *p* < 0.001). Conversely, those in the highest ADI quartile (most disadvantaged) were more likely to have a later-stage diagnosis compared to those with earlier stage diagnosis (41.7% compared to 14.1%; *p* < 0.001).

Racial and ethnic differences were also observed. Among those diagnosed at an earlier stage, 95.7% were non-Hispanic White, compared to 89.4% of those diagnosed at a later-stage (*p* < 0.001). Insurance type followed a similar pattern. Those with private insurance were more likely to have an early-stage diagnosis while those with public insurance were over-represented among later-stage diagnosis (*p* < 0.001). Additionally, those diagnosed at an earlier stage were more likely to live in urban areas and to be older (*p* < 0.01, *p* < 0.001).

### 3.3. Associations in Stage at Diagnosis by ADI, Race/Ethnicity, Insurance, Rurality, and Age

Table 2 presents the logistic regression results predicting a later-stage at diagnosis among individuals with melanoma. Model 1 shows that compared to non-Hispanic Whites, individuals in the “other race” category have over three times the odds of having a later-stage diagnosis (OR: 3.36, *p* = 0.002). Non-Hispanic Blacks also have higher odds of receiving later-stage diagnosis compared to non-Hispanic Whites, though this difference fails to reach statistical significance (OR: 1.77, *p* = 0.277).

Model 2 included ADI as a predictor and revealed that a one-unit increase in ADI was associated with a 30% increase in the odds of being diagnosed at a later-stage (OR: 1.30, *p* < 0.001). After adjusting for ADI, the racial differences seen in Model 1 were reduced, although the “other race” category still had higher odds of later-stage diagnosis compared to non-Hispanic Whites (OR: 2.51, *p* = 0.025).

Model 3 included the covariates gender, marital status, insurance, urbanicity, and age. ADI continues to be strongly associated with later-stage diagnosis such that for every one-unit increase in the ADI, the odds of being diagnosed with later-stage melanoma increased by 35% (OR: 1.35, *p* < 0.001). Additionally, male sex, public insurance, urban residence, and younger age were all significantly associated with higher odds of later-stage diagnosis.

**Figure 2:**
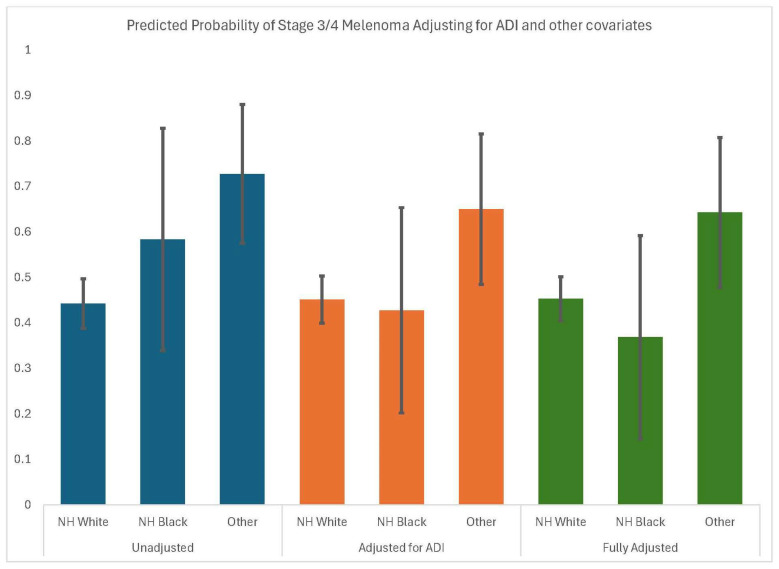
Predicted probability of stage 3/4 melanoma at diagnosis by race/ethnicity, adjusting for ADI and other covariates (age, gender, marital status, urbanicity, and health insurance).

Model 4 includes the interactions between race/ethnicity and ADI and are not significant, suggesting that the influence of ADI on stage is similar across race/ethnic groups. Figure 2 illustrates the predicted probability of later-stage diagnosis by race/ethnicity across the first three models. Although the difference between non-Hispanic Blacks and non-Hispanic Whites was never significant, we found that ADI accounted for much of the observed differences between these groups.

## 4. Discussion

### 4.1. Patient Demographics in Regional and National Context

Our study examined stage at melanoma diagnosis among 941 patients in the UAB Health System from 2010 to 2019. The cohort was predominantly non-Hispanic White and male, consistent with national melanoma incidence trends. SEER Registry data from 2010 to 2018 show that newly diagnosed cutaneous melanoma patients were 90.8% non-Hispanic White, 3.2% Hispanic, 0.4% non-Hispanic Black, and 58.4% male [11]. While our cohort was similar, it included a higher proportion of non-Hispanic Black patients and a lower proportion of Hispanic patients. This difference likely reflects Alabama’s demographic profile compared to national averages: according to 2010 Census data, Alabama’s population was 67% White alone, 26% Black or African American alone, and 4% Hispanic, versus the U.S. population at 64%, 12%, and 16%, respectively [30].

Nationally, 75.1% of melanomas are diagnosed at an early-stage, 14.3% at a late-stage, and 10.7% have tumors of unknown thickness [11]. In contrast, our study population had a substantially higher proportion of late-stage diagnoses. This may reflect regional disparities in healthcare access in Alabama as well as referral patterns to an academic medical center, where patients often present with more advanced disease.

### 4.2. Stage at Diagnosis by ADI, Race/Ethnicity, Insurance, Rurality, and Age

Our findings highlight significant associations between neighborhood socioeconomic disadvantages, as measured by the ADI, and the stage at which melanoma is diagnosed. Individuals residing in neighborhoods with higher ADI scores were more likely to be diagnosed at a later-stage, even after adjusting for key demographic and socioeconomic covariates. We found that a one-unit increase in ADI was associated with a 30–35% increase in the odds of late-stage diagnosis. This was also demonstrated through ADI quartiles, with the highest quartile showing a markedly greater proportion of late-stage diagnoses compared to the lowest quartile. These results are consistent with prior studies linking higher neighborhood deprivation with poorer melanoma outcomes across multiple measures [9,10,17,18,19,21,22]. This underscores the powerful impact of neighborhood socioeconomic conditions on health outcomes, particularly in cancer detection, and likely reflects complex barriers to early detection.

Importantly, racial and ethnic differences in stage at diagnosis were also evident, with non-Hispanic Whites being more likely to receive an early-stage diagnosis compared to other racial/ethnic groups. This aligns with national data and other studies showing disparities in melanoma detection and treatment among racial and ethnic minority groups [1,2,3]. However, these differences were attenuated after adjusting for ADI in our study, suggesting that socioeconomic disadvantage plays a critical role in shaping cancer outcomes across racial lines. Additionally, public insurance status, male sex, younger age, and urban residence were found to independently contribute to the likelihood of being diagnosed at a later-stage. These observations are also consistent with previous research studies [4,8,9,11,12,13].

Together, these findings suggest that addressing neighborhood-level socioeconomic disadvantages could be critical in reducing disparities in cancer outcomes and improving early detection efforts, particularly among underserved populations. By focusing on interventions at the community or neighborhood level, there is potential to mitigate known disparities in melanoma detection and ultimately improve survival rates.

### 4.3. Study Limitations

Several limitations should be considered when interpreting our findings. First, this study was conducted at a single academic medical center and did not capture all melanoma cases in the state of Alabama, which may limit the generalizability of our results. Patients at academic centers often differ demographically and clinically from the broader population, and our cohort included a higher proportion of late-stage diagnoses compared to national averages.

Second, the retrospective, cross-sectional design of the study precludes establishing causal or temporal relationships between neighborhood disadvantage and melanoma stage at diagnosis, although our findings highlight strong associations between these variables.

Third, the handling of missing data also presents a potential limitation, as entries with missing stage data were excluded, and duplicate records were removed to ensure data integrity. This may have resulted in the loss of some information and has the potential to introduce bias or reduce statistical power. Missing staging data could have occurred due to incomplete medical records, inconsistent reporting practices across providers, or cases where staging was not performed or documented at the time of diagnosis. Future studies should aim to collect a more comprehensive dataset, including melanoma cases across the entire state of Alabama or even across the southeastern United States. This would enhance the generalizability of the findings and further reveal how socioeconomic factors and racial disparities interact across geographical regions.

Finally, the analysis relied on patients’ addresses at the time of diagnosis, which may not fully reflect their long-term lived experiences or cumulative social vulnerability. We chose to use the patient’s address at the time of diagnosis to most accurately characterize their neighborhood vulnerability and socioeconomic context during this critical period of diagnosis.

### 4.4. Further Research

There are several avenues for further research. First, expanding the study cohort to include data from multiple institutions or population-based cancer registries would improve the generalizability of findings and allow for broader comparisons across geographic regions and healthcare settings. A larger, more diverse sample would also strengthen analyses of racial, ethnic, and socioeconomic disparities in melanoma and allow for a more nuanced examination of how these factors may differ or interact across diverse populations and contexts.

Secondly, although this study focused on stage at diagnosis as a proxy for melanoma outcomes given its association with survival [4,5,6,7], future studies may utilize survival data directly. Survival data may provide a more comprehensive measure of disparities by capturing the impact of socioeconomic disadvantage even after diagnosis, including treatment access, quality of care, and adherence.

Third, more detailed investigation into the mechanisms linking socioeconomic factors to cancer stage at diagnosis is needed. Potential pathways include barriers related to access such as distance to practicing dermatology providers, provider density and availability, transportation options, and healthcare infrastructure within disadvantaged areas. Other key factors to explore include cultural and language barriers that may hinder timely healthcare seeking or detection behavior, as well as the role of educational attainment in influencing awareness and access to preventative care. Understanding these mechanisms can inform the development of tailored interventions to improve early detection, particularly in underserved communities.

Additionally, prospective study design or linkage with longitudinal data would help clarify the causal and temporal relationships between neighborhood socioeconomic disadvantage and melanoma stage at diagnosis, addressing the inherent limitations in the retrospective, cross-sectional analyses described above.

Finally, the ADI could be utilized to develop and target specific interventions in areas with higher risk for late-stage melanoma diagnosis. The connection between late-stage diagnosis and higher ADI scores may allow public health efforts, community outreach, and education campaigns to concentrate resources in these areas to promote early cancer screening and increase access to healthcare services. Specifically, the development and implementation of mobile screening units, telemedicine initiatives, free or low-cost clinics, and community health partnerships may be particularly effective in these high-risk areas.

## 5. Conclusions

This study demonstrates that neighborhood-level socioeconomic disadvantage, as measured by the ADI, is significantly associated with later-stage melanoma diagnoses. Patients living in the most disadvantages neighborhoods had significantly higher late-stage diagnoses, independent of age, sex, insurance type, and urbanicity. Racial and ethnic differences in stage at diagnosis were also observed, with non-Hispanic White more likely to be diagnosed at an earlier stage; however, these differences were largely attenuated after accounting for neighborhood disadvantage. Additionally, public insurance, male sex, younger age, and urban residence were independently associated with late-stage melanoma. These findings underscore the importance of SDOH in melanoma outcomes. Addressing these disadvantages through targeted public health interventions could impact early detection, improve cancer outcomes, and reduce racial disparities seen in melanoma survival rates, particularly in vulnerable populations. Future research should expand to more diverse cohorts, incorporate longitudinal data, and may examine survival outcomes to further elucidate these disparities.

## Figures and Tables

**Table 1:** Characteristics of the study sample stratified by melanoma stage.

Descriptive Statistics of the Study Sample by Melanoma Stage										
	Full Sample (n = 941)			Stage 3/4 (n = 432)			Stage 1/2 (n = 509)			
	Mean/%	95% CI		Mean/%	95% CI		Mean/%	95% CI		
Stage 3/4	45.9	42.7	49.1							
ADI (continuous)	4.26	0.10		5.40	0.14		3.30	3.08	3.5	***
ADI Quartiles										
1 (Lowest)	24.9	22.1	27.6	11.8	8.8	14.9	36.0	31.8	40.1	***
2	24.1	21.4	26.9	21.8	17.9	25.7	26.1	22.3	30.0	***
3	24.2	21.5	27.0	24.8	20.7	28.9	23.8	20.1	27.5	***
4 (Highest)	26.8	23.9	29.6	41.7	37.0	46.3	14.1	11.1	17.2	***
Race/ethnicity										
NH White	92.8	91.1	94.4	89.4	86.4	92.3	95.7	93.9	97.5	***
NH Black	2.6	1.5	3.6	3.2	1.6	4.9	2.0	0.8	3.2	+
Other	4.7	3.3	6.0	7.4	4.9	9.9	2.4	1.0	3.7	**
Gender										
Female	37.0	33.9	40.1	33.8	29.3	38.3	39.7	35.4	44.0	
Male	63.0	59.9	66.1	66.2	61.7	70.7	60.3	56.0	64.6	
Marital Status										
Married	63.1	60.0	66.2	61.8	57.2	66.4	64.2	60.1	68.4	
Divorced/Sep	14.3	12.1	16.6	14.8	11.5	18.2	13.9	10.9	17.0	
Single	11.1	9.0	13.1	13.0	9.8	16.1	9.4	6.9	12.0	
Widowed	11.5	9.4	13.5	10.4	7.5	13.3	12.4	9.5	15.2	
Insurance										
Private	42.3	39.1	45.5	35.6	31.1	40.2	47.9	43.6	52.3	***
Public	53.9	50.7	57.1	59.0	54.4	63.7	49.5	45.2	53.9	***
None/other	3.8	2.6	5.1	5.3	3.2	7.4	2.6	1.2	3.9	*
Urban	56.0	52.8	59.2	50.2	45.5	55.0	60.9	56.7	65.2	**
Age	64.0	0.47		62.2	0.7		65.6	0.63		***

+ *p* < 0.1, * *p* < 0.05, ** *p* < 0.01, *** *p* < 0.001. Significance tests are between stage 1/2 and stage 3/4.

**Table 2:** Logistic regression models predicting Stage 3/4 among those diagnosed with melanoma. Model 1 includes race/ethnicity. Model 2 includes ADI. Model 3 includes age, gender, marital status, urbanicity, and health insurance.

Logistic Regression Predicting Stage 3/4 Among Those with Melanoma (Standard Errors Clustered at the Census Tract)																
	Model 1				Model 2				Model 3				Model 4			
	OR	*p*-Value	95% CI		OR	*p*-Value	95% CI		OR	*p*-Value	95% CI		OR	*p*-Value	95% CI	
Race/ethnicity																
(ref = NH White)																
NH Black	1.77	0.277	0.63	4.92	0.90	0.84	0.31	2.59	0.65	0.484	0.20	2.16	0.66	0.755	0.05	8.68
Other	3.36	0.002	1.55	7.32	2.51	0.025	1.12	5.62	2.53	0.030	1.10	5.85	1.25	0.813	0.20	7.68
ADI					1.30	<0.001	1.19	1.41	1.35	<0.001	1.24	1.48	1.35	<0.001	1.23	1.48
X NH Black													1.00	1.000	0.71	1.41
X Other													1.17	0.419	0.80	1.71
Gender																
(ref = Female)																
Male									1.47	0.036	1.03	2.10	1.46	0.037	1.02	2.09
Marital Status																
(ref = Married)																
Divorced/Sep									0.72	0.252	0.41	1.26	0.71	0.235	0.41	1.25
Single									0.94	0.843	0.53	1.68	0.93	0.811	0.52	1.66
Widowed									1.20	0.540	0.66	2.18	1.19	0.570	0.65	2.16
Insurance																
(ref = Private)																
Public									2.82	<0.001	1.83	4.33	2.82	<0.001	1.83	4.35
None/other									1.90	0.149	0.79	4.53	1.95	0.127	0.83	4.61
Urban									1.71	0.036	1.04	2.82	1.71	0.036	1.04	2.83
Age									0.96	<0.001	0.95	0.98	0.96	<0.001	0.95	0.98

## Data Availability

Data is available upon request.

## Abbreviations

The following abbreviations are used in this manuscript:

ADI	Area Deprivation Index
SDOH	Social determinants of health
SES	Socioeconomic status
ACS	American Community Survey
RUCA	Rural-Urban Commuting Area
